# Effect of an organizational change in a prehospital trauma care protocol and trauma transport directive in a large urban city: a before and after study

**DOI:** 10.1186/s13049-016-0218-3

**Published:** 2016-03-09

**Authors:** Rebecka Rubenson Wahlin, Sari Ponzer, Markus B. Skrifvars, Hans Morten Lossius, Maaret Castrén

**Affiliations:** Department of Clinical Science and Education, Södersjukhuset, Karolinska Institutet, Stockholm, SE-118 83 Sweden; Division of Intensive Care, Department of Anesthesiology, Intensive Care and Pain Medicine, University of Helsinki and Helsinki University Hospital, Helsinki, FI-00029 HUS Finland; Field of Prehospital Critical Care, Network for Medical Sciences, University of Stavanger, Kjell Arholmsgate 41, Stavanger, NO 4036 Norway; Department of Emergency Medicine and Services, University of Helsinki and Helsinki University Hospital, Helsinki, FI-00029 HUS Finland; Department of Anesthesia and Intensive Care, Södersjukhuset, Stockholm, SE-118 83 Sweden

**Keywords:** Trauma, Injury, Late adolescent and adult trauma care, Prehospital care, Emergency services

## Abstract

**Background:**

Trauma systems and regionalized trauma care have been shown to improve outcome in severely injured trauma patients. The aim of this study was to evaluate the implementation of a prehospital trauma care protocol and transport directive, and to determine its effects on the number of primary admissions and secondary trauma transfers in a large Scandinavian city.

**Methods:**

We performed a retrospective observational study based on local trauma registries and hospital and ambulance records in Stockholm County; patients > 15 years of age with an Injury Severity Score (ISS) > 15 transported to any emergency care hospitals in the Stockholm area were included for the years 2006 and 2008. We also included secondary transferred patients to the regional trauma center during 2006, 2008, and 2013.

**Results:**

A total of 693 primarily admitted trauma patients were included for the years 2006 and 2008. For the years 2006, 2008 and 2013, we included 114 secondarily transported trauma patients. The number of primary patient transports to the trauma center increased during the years by 20.2 %, (*p* < 0.001); patients primarily transported to the trauma center had a significantly higher Injury Severity Score in 2008 than in 2006, and the number of patients transported secondarily to the trauma center in 2006 was higher compared to 2008 and to 2013 (*p* < 0.001, all 3 years).

**Discussion:**

Our data indicate that implementation of a prehospital trauma care protocol may have an effect on transportation of severely injured trauma patients. A decrease in secondarily transported trauma patients to the regional trauma center was noted after 1 year and persisted at 7 years after the organizational change. Patients primarily admitted to the trauma center after the change had more severe injuries than patients transported to other emergency hospitals in the area even if 20 % of patients were not admitted primarily to a trauma center. This does not imply that the transport directives or the criteria were not followed but rather reveals the difficulties and uncertainties of field triage.

**Conclusions:**

With the introduction of a prehospital trauma transport directive in a large urban city, an increase in patients transported to the regional trauma center and a decrease in secondary transfers were detected, but a considerable number of severely injured patients were still transported to local hospitals.

## Background

Injury is a major cause of death among individuals under 45 years of age worldwide [1]. Trauma systems and regionalized trauma care have been shown to have an impact on the outcome of trauma [2–8]. The term “trauma system” refers to a way of organizing trauma care in a specific region and includes strategies for injury prevention, protocols for prehospital assessment, ambulance transport directives, a trauma center, post-trauma care, and follow-up [6, 9].

The assessment triage is an essential part of a trauma system and is aimed at determining the need for specialist trauma care. Previous studies have shown that critically injured trauma patients benefit from treatment at a trauma center [2, 5, 10]. Improved outcomes such as mortality, morbidity, shorter intensive care periods, and a shorter total length of stay (LOS) have been seen after implementation of trauma systems [11–14]. Both overtriage and undertriage may have an unfavorable impact on the system. Undertriage can result in an increase in secondary (i.e. inter-hospital) transfers and suboptimal care for severely injured patients and may also result in increased mortality [15]. An Australian study showed that older patients with fall injuries were more likely to be undertriaged [16]. On the other hand, overtriage can result in overcrowding of trauma centers and cost ineffectiveness [17, 18]. Secondary transfer of patients might have negative impact on outcomes with an increased risk for mortality [19], but the evidence is inconclusive [20]. Studies have also suggested that implementation of a trauma system can be cost-effective [21–23]. Most previous studies on trauma systems have focused on exclusive trauma systems where the patient is transported to the region’s designated trauma center. This has served the severely injured patient well, particularly in the urban regions, but has been harder to implement in non-urban settings. The focus has then shifted towards more inclusive and integrated systems where both trauma centers and non-trauma hospitals are important for delivering trauma care to the region regardless of habitation status and have the ability to match the patients’ injury with the right level of care [9, 24]. Some recent studies from Canada have focused on integrated systems [24, 25], but more research is needed to evaluate these systems in terms of performance and cost effectiveness. The majority of the studies on trauma systems and trauma outcomes have been conducted outside of Europe [26] and Scandinavia [27] therefore the results may not be applicable to our slightly different trauma systems.

The Emergency Medical Services (EMS) in Stockholm implemented a new prehospital trauma care protocol on July 1st, 2007, which included a new prehospital regional trauma transport directive to transport of the most severely injured patients to Karolinska University Hospital at Solna, the single trauma center in the Stockholm area. Prior to this directive, all trauma patients, regardless of injury type or severity, were transported to the nearest hospital without taking into consideration the receiving hospital’s capability in terms of skills, staffing, training, and competence in caring for severely or multiple-injured patients [28].

The aim of this study was to evaluate the effect on patient flow to the trauma center after implementation of **a** prehospital trauma care protocol in a large Scandinavian city. The hypothesis was that the trauma care protocol and trauma transport directive would steer critically injured patients directly to the trauma center (primary outcome) and reduce the number of secondary transfers (secondary outcome).

## Methods

We performed a retrospective register study comparing the periods January 1st–December 31st, 2006, and January 1st–December 31st, 2008, i.e., 1 year before and 1 year after the organizational changes were made. To evaluate changes over time of the system, the period from January 1st to December 31st, 2013, was compared to the years 2006 and 2008 regarding secondarily transferred patients.

### Setting

This study was conducted in the Stockholm County Council (SCC) area in Sweden. This area comprises approximately 2 million inhabitants, which is about one fifth of the Swedish population, and consists of 26 municipalities with an area of 6519 square kilometers, including an archipelago of approximately 30,000 islands of various sizes [29]. The SCC is responsible for all healthcare provided in the region, including prehospital trauma care.

In the SCC area, there are seven emergency hospitals, but only one can be regarded as a level-1 trauma center according to the American College of Surgeons’ criteria, namely Karolinska University Hospital, Solna. Distances from the other emergency hospitals to the trauma center vary between 5 km and 67 km.

The acute care hospitals’ emergency departments (EDs) used a variety of triage systems in 2006, where patients were categorized as triage level 1–4/5, depending on the hospital system. A more uniform system was implemented in all EDs in 2008, where all patients were triaged into 5 categories: red = 1, orange = 2, yellow = 3, green = 4, and blue = no triage needed. The same system was still in use in 2013.

The EMS in the studied area is run by both SCC and private companies, all of them governmental funded. During 2006 and 2008, the EMS constituted 55 ground ambulances, one ambulance helicopter, one mobile intensive care unit (MICU), and three rapid response cars operating in the area (57). A rapid response car was called to severe accidents as second tier providing early advanced resuscitation assisting the crew of the regular ambulances. In 2008, it became mandatory to man the ambulances with a specialist nurse (prehospital emergency medicine, anesthesiology or intensive care). From 2008 to 2013, the number of ground ambulances had increased to 61, but the staffing and training were identical to 2008 (Table 1).

**Table 1:** EMS units and staffing, 2006, 2008 and 2013

EMS unit	2006	2008 and 2013
Ambulance	EMT and registered nurse	EMT and specialist nurse
Rapid response car 1 and 2	EMT and nurse anesthetist	EMT and nurse anesthetist
Rapid response car 3		EMT and anesthesiologist
Helicopter	EMT, nurse anesthetist, anesthesiologist and pilot.	EMT, nurse anesthetist and pilot.
Mobile intensive care unit (MICU)	EMT and specialist nurse	EMT and specialist nurse

### Study population

In the first two study periods (2006 and 2008) we included adult trauma patients (>15 years of age) with an Injury Severity Score (ISS) > 15, transported by ground or helicopter ambulance to any of the seven emergency hospitals in the Stockholm area. For 2013 we included only adult trauma patients (>15 years of age) with ISS > 15 secondarily transferred to the Karolinska University Hospital Trauma Center within 24 h from the injury. This secondary transfer data from 2013 was included as a “marker” how the system has maturated over the years since it was introduced.

Patients with traumatic cardiac arrest and ongoing cardiopulmonary resuscitation (CPR) during transport to hospital were included even if no return of spontaneous circulation (ROSC) occurred during transport. We excluded trauma patients who were declared clinically dead on-scene for whom no resuscitative measures were taken, patients admitted to the reporting hospital > 24 h after the trauma, and patients suffering asphyxia due to drowning.

Primary admissions were defined as patients transported directly from the scene to trauma center within 24 h after trauma; secondary transfers were defined as patients transferred from any other hospital within 24 h after trauma to the trauma center.

Excluded secondary transfers were those involving patients from another county for specialist care and/or transfers > 24 h after the initial admission to a local hospital.

We included variables according to the Utstein template for major trauma [30]: age, gender, dominating type of injury, injury mechanism, intentional injury, systolic blood pressure at arrival on scene, respiratory rate at arrival on scene and the Glasgow Coma Scale (GCS) score [31] at arrival on scene, prehospital cardiac arrest, type of prehospital transportation, and inter-hospital transfers (i.e. secondary transfers). In addition, the following variables were added for the purpose of this study: prehospital triage level, prehospital priority and the Injury Severity Score (ISS) [32].

### The prehospital trauma care protocol

The prehospital guidelines for trauma triage before July 1st, 2007, included only anatomical and descriptive criteria concerning the injury mechanism and were used to alert the receiving hospital for an incoming trauma patient. There was no actual protocol and the triage was based on the EMS crew’s clinical observation of the patient.

**Fig. 1:**
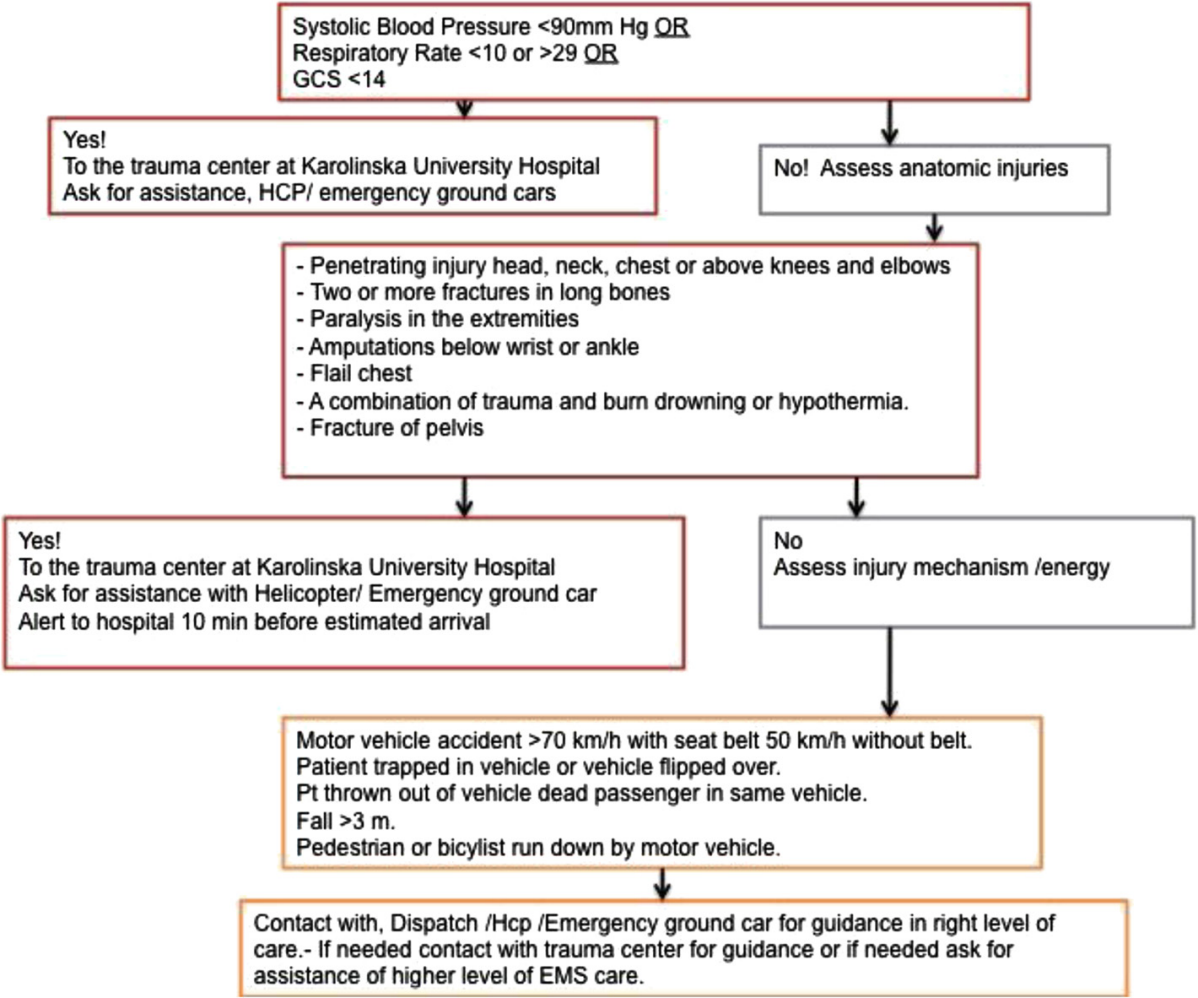
Prehospital trauma care protocol from 2007 to 2011

The triage protocol implemented in 2007 included vital parameters, i.e., whether the systolic blood pressure was less than 90 mmHg, the respiratory rate below 10 or higher than 29, or the Glascow Coma Scale (GCS) score was less than 14, and stated that the trauma patient should be transported to the trauma center directly regardless of bypassing the nearest hospital. If the patient had normal vital parameters, the anatomical injuries should be assessed and the trauma mechanism should be regarded as part of the criteria (Fig. 1).

### Data collection

Data for 2006 and 2008 were collected from trauma registry “Kvalitet i Trauma Sjukvården”, KVITTRA/QUITC (version 14.0) at the Karolinska University Hospital (Solna and Huddinge). The data on secondary transfers for years 2006, 2008 and 2013 were collected from the Trauma Registry at the Trauma Center.

Data from the second largest hospital in the area, Södersjukhuset, were collected from the trauma registry TRAUMAREG (version TraumaSys 2000–2001, version 1.1.), and completed by data from the hospital’s digital patient registration system (Pasett-DRG, version 1.61) regarding length of stay. Data from the four other local hospitals were retrieved from the digital patient records (Take Care, Melior, and Cambio Cosmic), and trauma patients identified from emergency department records. Since no uniform reporting system or term for admitted trauma patients existed, the records were examined manually. Records of all patients transported by ambulance or helicopter to the surgical or orthopedic sections of the emergency departments and with a traumatic injury mechanism, and ED priority level of 1 or 2 and/or admitted to a hospital ward were examined for injury severity. In addition, all patients with suspected head trauma or patients directly admitted to the ICU or operating room from the ED were scanned, regardless of the priority given at the ED. In one hospital it was not possible to obtain all hospital admission records and therefore only pre-alert trauma patients were examined for eligibility. Prehospital data were collected from the digital ambulance records (CAK-net) used by all ambulance caregivers in SCC. The data collection is shown in Table 1.

The patients were identified through their unique Swedish social security number. Foreign patients received a temporary number given by the admitting hospital, making it possible to track these patients between hospitals in case of a secondary transfer. For patients included from the four hospitals without trauma registries the Abbreviated Injury Score (AIS, version 2005) [33] and the Injury Severity Score (ISS) [32] were calculated by trained trauma registry personnel and by one of the authors (RRW).

### Ethical approval

The study received ethical approval by the Regional Ethics Committee in Stockholm (Reg. Nos: 2007/1113-31; 2010/1979-32, 2013/1718-32 and 2014/691-32).

### Statistics

Continuous variables are presented with the median, mean and interquartile range (IQR), range and categorical variables, with the count (n) and percentage (%). Since none of the variables were normally distributed, the Mann–Whitney *U* test was used for continuous data and chi-square for categorical data. Data were statistically analysed using IBM SPSS Statistics, version 22.0.0.0. The statistical level of significance was set to *p* < 0.05.

## Results

Three hundred and ten patients were included from 2006, and 383 patients from 2008 (Table 2). The majority of the injuries were due to blunt trauma and the predominant injury mechanism was traffic-related during both years. No difference in gender or age distribution was noted. Table 3 shows the characteristics of the study population. The median ISS was significantly lower in 2006 than in 2008 (20 and 24, respectively, *p* < 0.001). The priority of ambulance transports did not differ between the years, nor did the number of prehospital traumatic cardiac arrests (Table 3).

**Table 2:** Number of patients retrieved from different hospitals’ data sources

Hospital	2006		2008	
	Number of patients before inclusion	Number of patients included with ISS > 15	Number of patients before inclusion	Number of Patients included with ISS > 15
Trauma Center	999	189	1717	307
Hospital 2	293	18	118	1
Hospital 3	214	78	136	46
Hospital 4	366	7	337	12
Hospital 5	31	2	91	1
Hospital 6	1025	10	396	9
Hospital 7	389	6	424	7

Hospitals 1–3, data from trauma registries; Hospitals 4–7, data from manual collections

**Table 3:** Patient characteristics

Variables	2006 *n* = 310 (%)	2008 *n* = 383 (%)	*p*-value* sign
Gender			0.938
Male	225 (72.6)	279 (72.8)	
Female	85 (27.4)	104 (27.2)	
Age	50 (30–66)	45 (28–69)	0.514
Injury Severity Score (ISS)	20 (17–26)	24 (18–30)	<0.001*
Hospital length of stay (LOS)	6.9 (1.5–16.4)	7.7 (2.8–17.7)	0.137
Total prehospital time	40 (29–51)	42 (32–53)	0.085
Prehospital on-scene time	16 (11–25)	17 (12–24)	0.996
Predominant type of injury			0.221
Blunt	290 (93.9)	350 (91.4)	
Penetrating	19 (6.1)	33 (8.6)	
Missing	1 (0.3)		
Injury mechanism			0.754
Traffic	127 (41.1)	152 (39.8)	
Low-energy fall	65 (21.0)	71 (18.6)	
High-energy fall	70(22.7)	95 (24.9)	
Other	47 (15.2)	64 (16.8)	
Missing	1 (0.3)	1 (0.2)	
Type of injury			0.859
Accident	257 (84.3)	316(83.2)	
Self-inflicted	19 (6.2)	23 (6.1)	
Assault	29 (9.5)	41 (10.8)	
Missing	5 (1,6)	3 (0.8)	
Prehospital priority			0.925
Priority 1	253 (84.1)	310 (83.8)	
Priority > 1	48 (15.9)	60 (16.2)	
Missing	9 (2.9)	13 (3.4)	
Systolic blood pressure RTS category,			0.954
Systolic blood pressure RTS 4	242 (80.1)	292 (78.1)	
Systolic blood pressure RTS 0–3	60 (19.9)	82 (21.9)	
Missing	19 (6.1)	17 (4.4)	
Respiratory rate RTS category			0.241
Respiratory rate RTS 4	247 (84.3)	311 (85.7)	
Respiratory rate RTS 0–3	46 (15.7)	52 (14.3)	
Missing	17 (5.5)	20 (5.2)	
Glasgow Coma Scale RTS category			0.514
Glasgow Coma Scale RTS 3–4	246 (84.5)	310 (84.7)	
Glasgow Coma Scale RTS 0–2	45 (15.5)	56 (15.3)	
Missing	8 (2.5)	9 (2.3)	
Prehospital cardiac arrest (yes)	8 (2.6)	15 (3.9)	0.336
Missing	5 (1.6)	4 (1.0)	
30-day mortality (dead)	57 (19.1)	68 (18.1)	0.742
Missing	17 (4.9)	2 (0.5)	

Categorical values presented with crude numbers and percent and continuous variables with median and IQR

* p < 0.05

**Fig. 2:**
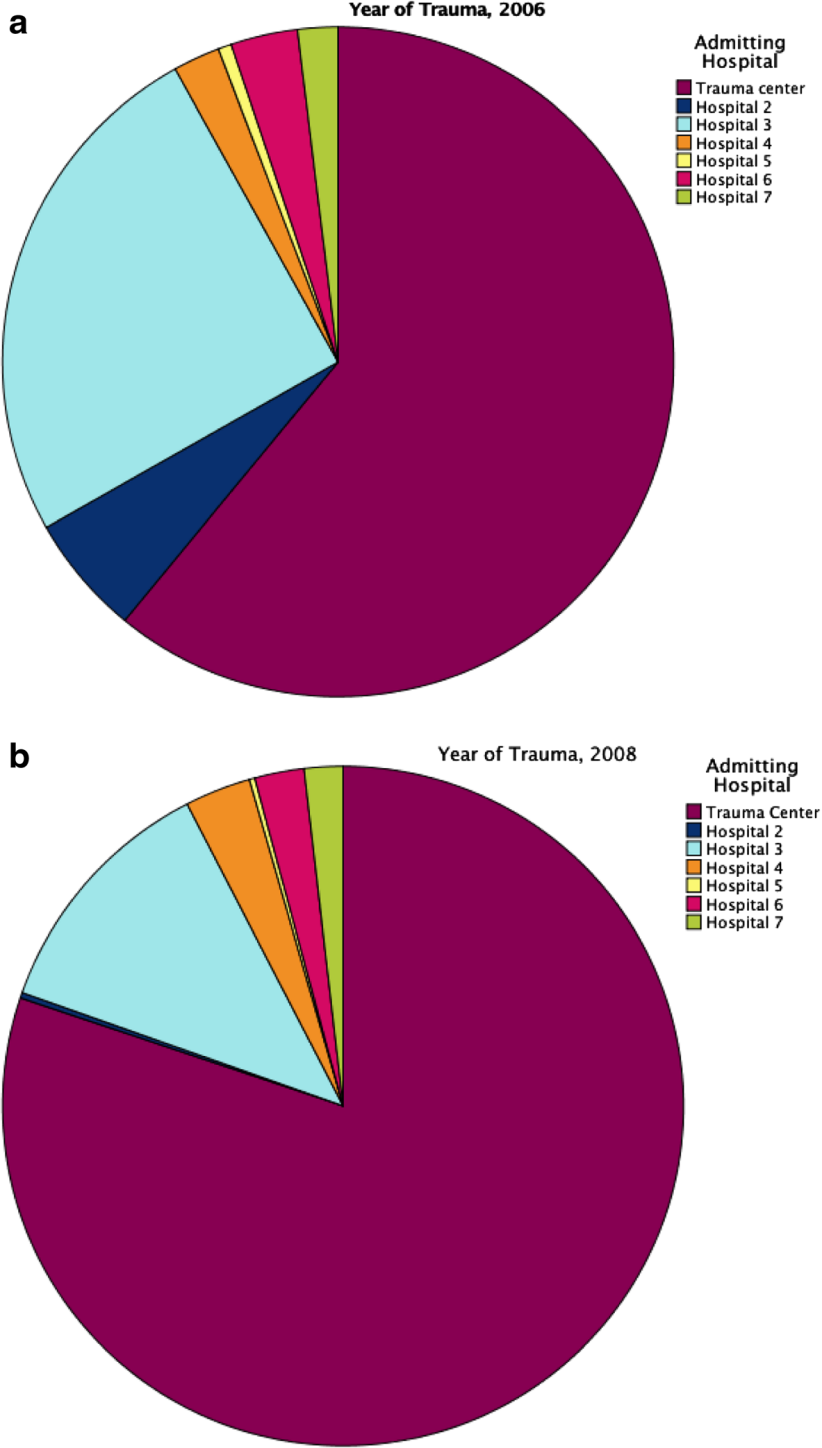
Distribution of trauma patients between hospitals in 2006 (**a**) and in 2008 (**b**)

The number of patients transported to the trauma center increased between the years by 20.2 %, i.e., from *n* = 189 patients to *n* = 307, *p* < 0.001. Table 4, as well as Fig. 2, displays both the distribution of patients transported directly to the trauma center and transports to the non-trauma center hospitals. Patients transported to the trauma center had a significantly higher ISS score in 2008 than in 2006 (*p* < 0.001) (Table 4).

**Table 4:** Patient distribution between trauma center and all other hospitals years 2006 and 2008

Hospital	2006 (*n* = 310)		2008 (*n* = 383)	
	n (%)	ISS median (IQR)*	n (%)	ISS median (IQR)*
Trauma Center	189 (61.0)	21 (17.27)	307 (80.2)	25 (19.30)
Non-Trauma Center	121 (39.0)	18 (17.25)	76 (19.8)	20 (17.26)

Number of patients and percent, ISS median and IQR, 2006, *p* = 0.015^*^, and 2008, *p* < 0.001^*^

Secondary transfers decreased significantly between years 2006 (*n* = 47) and 2008 (*n* = 32) (*p* < 0.001), but no further decrease was noted in 2013 (*n* = 35). There were no significant differences in age or ISS for the secondary transferred patients between the studied periods and the majority of the patients were males during all three studied periods.

## Discussion

The results of this study indicates that implementation of a prehospital trauma care protocol in a large urban city may have an effect on primary transportation rates of severely injured trauma patients. A decrease in secondary transfers to the regional trauma center was seen after 1 year and persisted 7 years after the organizational changes. Patients primarily admitted to the trauma center after the change were more severely injured than patients transported to other emergency hospitals in the area.

A study from New South Wales in Australia evaluating a modified version of the ACS-COT prehospital trauma triage protocol reported that approximately a quarter of the patients injured in an urban area were transported to a non-trauma hospital [34]. This study is particularly interesting since the trauma triage protocol they implemented has similarities to the protocol we have studied. However, in our study, we focused on evaluating the ability of the new trauma system to direct severe trauma patients to the trauma center and did not evaluate the actual performance of the protocol criteria. Therefore, with almost 20 % of the patients still not being transported direct to the trauma center in 2008, there is a possibility that the performance of the new triage protocol was not optimal.

Demetriades [35] and Meisler et al. have reported that early transfer to a trauma center might improve survival [36]. Further, Nirula et al. have concluded that secondary transfers from a non-trauma hospital to a trauma center increases the risk of mortality [7] as compared to primary admissions. All patients in our sample were severely traumatized (ISS score > 15), which makes it is fair to assume that the majority of these patients would benefit from trauma center care.

Cudnik et al. reported improved survival and better functional outcomes for injured patients transported directly to a level I trauma center, compared to those taken to a level II center [5]. They also showed that patients with an intracranial injury and/or skull fracture, as well as patients with pelvic fractures, had a better outcome when treated at a level I center. The same was demonstrated by Demetriades et al. [35] and Garwe et al., who reported a survival benefit for patients transferred to a level I facility from level III or IV facilities [10]. Some studies have reported transportation of severely injured patients to non-trauma centers with proportions between 30 % and 60 % [23, 37], but others have reported no survival benefit from direct transportation to a trauma center [38–41]. However, Haas et al. [22] have pointed out that the value of these studies is limited due to the fact that they were based on data from trauma registries where no account was taken of patients who died before transfer. They showed that for inter-facility transfers that included patients who died while waiting for transfer, the mortality rate increased by 25 % and concluded that undertriage was associated with higher mortality and that primary admissions to a trauma center was beneficial. In our study 20 % of patients were not admitted primarily to a trauma center. However, we believe that this fact does not mean that the transport directives or the criteria were not followed since this study did was not designed to evaluate the criteria as such. We believe that the results mainly imply the difficulties and uncertainties of field triage, an area where further research is needed.

Our study has a retrospective design with the majority of the data collected from trauma registries. There is a possibility of missing cases, which might be a limitation. However, trauma documentation was a prioritized area for the hospitals during the periods included in our study, with educated trauma registrars being responsible for data collection. During the studied years, some changes were made in the EMS system. In 2008 and 2013, the ambulance crews consisted of at least one nurse with specialist training, which was not the case in 2006. A new trauma care protocol was implemented at the receiving hospitals’ EDs between the data collection years, with triage levels corresponding to the new trauma protocol in the prehospital system, while in 2006 all EDs still had their own independent triage systems, a fact that made it more difficult to compare triage levels between hospitals. One of the hospitals did not have a system that allowed us to retrieve full admission records for the years studied, which made it impossible to scan triage levels for injury severity among all patients admitted to the surgical and orthopedic sections of the ED, or for head traumas.

Despite these limitations, our study is the first to evaluate the effect of a new trauma care protocol and transport directive for trauma patients in our region. It is necessary for a trauma system to mature for several years and further evaluation of the system will be needed. The goal of a trauma system is to get the right patient to the right facility at the right time. Studying how a trauma system works is the key to achieve this.

## Conclusions

This study focusing on the effects of implementing an improved trauma care protocol and a trauma transport directive in a large urban city indicates an increased frequency of patients primarily admitted to the regional trauma center and a decrease in secondary transfers. Nonetheless, almost 20 % of the severely injured patients were still transported to the emergency hospitals after implementation. Based on these findings, our system of regionalized trauma care and expedite immediate trauma center admission will be further improved.

## Abbreviations

ACOS: American College of Surgeons
AIS: abbreviated injury scale
CPR: cardiopulmonary resuscitation
ED: emergency department
EMS: emergency medical services
EMT: emergency medical technicians
GCS: glasgow coma scale
ICU: intensive care unit
IQR: interquartile range
ISS: injury severity score
KSS: Karolinska University Hospital in Solna
KVITTRA/QUITC: quality in trauma care
LOS: length of stay (days)
MICU: mobile intensive care unit
OR: Odds ratio
CI: confidence interval
ROSC: return of spontaneous circulation
RTS: revised trauma score
RR: respiratory rate
SBP: systolic blood pressure
SCC: Stockholm County Council
